# Investigating the potential clinical benefit of Selumetinib in resensitising advanced iodine refractory differentiated thyroid cancer to radioiodine therapy (SEL-I-METRY): protocol for a multicentre UK single arm phase II trial

**DOI:** 10.1186/s12885-019-5541-4

**Published:** 2019-06-14

**Authors:** Sarah R. Brown, Andrew Hall, Hannah L. Buckley, Louise Flanagan, David Gonzalez de Castro, Kate Farnell, Laura Moss, Rebecca Gregory, Kate Newbold, Yong Du, Glenn Flux, Jonathan Wadsley

**Affiliations:** 1 Leeds Institute of Clinical Trial Research, University of Leeds, Leeds, LS2 9JT UK; 2 Centre for Cancer Research and Cell Biology, Queen’s University Belfast, Belfast, BT9 7BL Northern Ireland, UK; 3 Butterfly Thyroid Cancer Trust, NCCC Freeman Hospital, Newcastle, NE39 2PU UK; 4 Velindre Cancer Centre, Cardiff, CF14 2TL UK; 5 Joint Department of Physics, The Royal Marsden NHS Foundation Trust and Institute of Cancer Research, Sutton, SM2 5PT UK; 6 The Royal Marsden NHS Foundation Trust, Sutton, SM2 5PT UK; 7 Department of Nuclear Medicine, The Royal Marsden NHS Foundation Trust, Sutton, SM2 5PT UK; 8 Weston Park Hospital, Sheffield, S10 2SJ UK

**Keywords:** Iodine refractory differentiated thyroid cancer, MEK inhibitor, Selumetinib, Radioiodoine therapy, Phase II, Dosimetry

## Abstract

**Background:**

Thyroid cancer is the most common endocrine malignancy. Some advanced disease is, or becomes, resistant to radioactive iodine therapy (refractory disease); this holds poor prognosis of 10% 10-year overall survival. Whilst Sorafenib and Lenvatinib are now licenced for the treatment of progressive iodine refractory thyroid cancer, these treatments require continuing treatment and can be associated with significant toxicity.

Evidence from a pilot study has demonstrated feasibility of Selumetinib to allow the reintroduction of I-131 therapy; this larger, multicentre study is required to demonstrate the broader clinical impact of this approach before progression to a confirmatory trial.

**Methods:**

SEL-I-METRY is a UK, single-arm, multi-centre, two-stage phase II trial. Participants with locally advanced or metastatic differentiated thyroid cancer with at least one measureable lesion and iodine refractory disease will be recruited from eight NHS Hospitals and treated with four-weeks of oral Selumetinib and assessed for sufficient I-123 uptake (defined as any uptake in a lesion with no previous uptake or 30% or greater increase in uptake). Those with sufficient uptake will be treated with I-131 and followed for clinical outcomes. Radiation absorbed doses will be predicted from I-123 SPECT/CT and verified from scans following the therapy. Sixty patients will be recruited to assess the primary objective of whether the treatment schedule leads to increased progression-free survival compared to historical control data.

**Discussion:**

The SEL-I-METRY trial will investigate the effect of Selumetinib followed by I-131 therapy on progression-free survival in radioiodine refractory patients with differentiated thyroid cancer showing increased radioiodine uptake following initial treatment with Selumetinib. In addition, information on toxicity and dosimetry will be collected. This study presents an unprecedented opportunity to investigate the role of lesional dosimetry in molecular radiotherapy, leading to greater personalisation of therapy. To date this has been a neglected area of research. The findings of this trial will be useful to healthcare professionals and patients alike to determine whether further study of this agent is warranted. It is hoped that the development of the infrastructure to deliver a multicentre trial involving molecular radiotherapy dosimetry will lead to further trials in this field.

**Trial registration:**

SEL-I-METRY is registered under ISRCTN17468602, 02/12/2015.

**Electronic supplementary material:**

The online version of this article (10.1186/s12885-019-5541-4) contains supplementary material, which is available to authorized users.

## Background

Thyroid cancer is the most common endocrine malignancy, but remains relatively rare despite incidence doubling since the 1990s. In 2014, 3400 people in the United Kingdom (UK) were diagnosed with thyroid cancer and in 2014 approximately 370 patients died from the disease [1].

### Current treatment

A combination of surgery, radioactive iodine (RAI) therapy and long-term thyroid stimulating hormone (TSH) suppression cures many patients with differentiated thyroid cancer, namely papillary and follicular cancers. However around 10% of patients develop advanced disease which will eventually become resistant to therapy. Prognosis for these patients is poor with a 10-year overall survival rate of 10% compared with 56% in patients with RAI avid metastases [2].

### Treatment for radioiodine refractory disease

Attempts have been made in the past to restore RAI avidity with lithium carbonate [3] and retinoic acid [4], but results have been disappointing and have failed to show clear clinical benefit.

More recently a number of multi-targeted kinase inhibitors have been tested; two phase III trials have shown significant improvements in progression free survival (PFS) with Sorafenib and Lenvatinib when compared with placebo [5, 6]. While these improvements are certainly to be welcomed, both agents cause toxicities, including fatigue, diarrhoea, hand-foot syndrome, weight loss and hypertension. Since they are taken continuously to disease progression this can have a significant impact on patients’ quality of life.

### MEK and radioiodine refractory disease

Mutations of RET, RAS or BRAF are present in approximately 70% of papillary thyroid cancers [7]. These oncogenes are part of the mitogen-activated protein kinase (MAPKinase) signalling pathway. Preclinical data suggest that activation of this pathway is critical to tumour initiation and transformation, and also results in reduced expression of genes involved in iodide metabolism, in particular the sodium iodide symporter (NIS) which is responsible for the uptake of iodine by thyroid cells. For example, papillary thyroid cancers with BRAF mutations are associated with reduced expression of key genes involved in iodine metabolism, including NIS [8].

In vitro work with rat thyroid PCCL3 cells with induced BRAF mutations has shown that treating the cells with a MEK inhibitor restores expression of TSH receptors, NIS and thyroglobulin, indicating that the effects of BRAF mutation to reduce NIS expression are potentially reversible [9].

### Selumetinib

Selumetinib is an oral, potent and highly selective, allosteric MEK1/2 inhibitor. MEK1/2 are critical components of the MAPKinase pathway. This pathway is frequently dysregulated due to activating mutations in various oncogenes, including RAS or RAF. Inhibition of MEK, which lies downstream of these targets, blocks inappropriate signal transduction offering a promising therapeutic strategy. Selumetinib is not currently approved for use in any clinical indications in humans, but phase I and II trials have been conducted in pancreatic cancer [10], melanoma [11–16], non-small cell lung cancer [17–19] and differentiated thyroid cancer [20–22] and it has recently been designated orphan drug status for treatment of differentiated thyroid cancer in the United States. The drug has been found to have an acceptable side effect profile; most frequently reported side effects include diarrhoea, nausea and vomiting, dyspnoea, blurred vision, tiredness and acneiform rash.

A previously reported pilot study [21] tested the use of Selumetinib 75 mg twice daily for four weeks in patients with radioiodine refractory differentiated thyroid cancer. All participants who started therapy were able to complete the full prescribed course of Selumetinib without dose reductions or delays. No toxic effects of Grade 3 or above were noted. Of 20 participants evaluated, Selumetinib increased radioiodine uptake in 12 (60%). Eight of these 12 achieved sufficient increased uptake for further radioiodine therapy to be viable (67%). Of the eight participants treated with radioiodine, five (63%) had partial responses and three (38%) had stable disease. All participants had decreases in serum thyroglobulin level.

### Lesional dosimetry

Standard practice in the UK, endorsed by recently published guidelines [23] is to prescribe an empirical activity, typically 3.7–7.4GBq iodine-131 (I-131) to patients with metastatic disease. It is well recognised that this leads to a wide range of absorbed dose delivered to tumour targets and organs at risk, and therefore very likely to lead to widely divergent outcomes. This is in stark contrast to modern external-beam radiotherapy practice where absorbed dose delivered to target volumes and to nearby organs at risk is prescribed and can be accurately measured. There have been calls to address this issue [24]. There is evidence that the absorbed dose delivered to thyroid remnants correlates with response for patients undergoing radioiodine ablation [25] and this study will investigate a similar relationship for patients with locally advanced or metastatic disease.

The previously described pilot study [21] used pre-treatment iodine-124 (I-124) positron emission tomography (PET) to estimate absorbed dose to individual lesions. A minimum lesion absorbed dose of 20Gy was chosen as a cut off for proceeding with further I-131 therapy. The choice of cut off used in this pilot does not appear to have been made on the basis of any published data. Additionally the absorbed doses were not verified after treatment, unlike in this study.

### SEL-I-METRY trial

The SEL-I-METRY trial has been designed to demonstrate the broader clinical impact of the use of Selumetinib therapy to re-sensitise radioiodine refractory thyroid cancer patients to treatment with radioiodine. The trial will address the dosimetry of I-131 therapy to investigate the relationship between the absorbed dose delivered and the clinical benefit from therapy. In addition the extent to which the therapeutic activity of I-131 can be individualised from the absorbed doses predicted in a pre-therapy tracer study will be analysed using dosimetric calculations. It will also explore possible biomarkers for response to this strategy, including measurement of post I-131 protein bound iodine (PBI) and molecular markers both in tumour tissue and circulating cell-free DNA.

## Methods

### Design

SEL-I-METRY is a UK, single arm, multi-centre, two-stage phase II proof of concept trial investigating the use of Selumetinib to resensitise iodine refractory patients with differentiated thyroid cancer. To ensure at least 38 participants are treated with I-131, approximately 60 participants will be recruited from eight NHS Hospitals. Participants will receive 75 mg of Selumetinib orally twice daily for four weeks (dose may be reduced due to adverse events) and will then be assessed for evidence of an increase in radioiodine uptake via iodine-123 (I-123) single photon emission computed tomography/computed tomography (SPECT/CT); those showing sufficient uptake will then receive I-131 therapy (further details on patient imaging and dosimetry can be found in Additional file 1). A previous pilot study [21] used I-124 sodium iodide (NaI) although there is currently no commercially available source of this isotope in the UK. This trial therefore elected to use I-123 as an alternative to determine changes in radioiodine uptake following Selumetinib therapy, and to predict likely lesional absorbed dose in participants proceeding to I-131 therapy. The use of I-123 as opposed to I-124 is not thought to pose any significant risk to the current trial; whilst theoretically there may be advantages to using I-124, there are no known clinical comparator studies between the two techniques and a pragmatic decision to use I-123 was deemed reasonable by the trial team. An early stopping rule is incorporated in the case where none of the first 10 patients treated with Selumetinib show sufficient iodine uptake to progress to I-131 therapy; should this happen the trial may be discontinued. The study protocol and this manuscript have been written in accordance with Standard Protocol Items: Recommendations for Interventional Trials (SPIRIT) guidelines [26] (checklist included as an Additional file 2).

### Ethical approval

The trial has received ethics approval from the NHS National Research Ethics Service (NRES) East Midlands-Leicester South (15/EM/0455).

### Primary objective

This trial aims to assess the efficacy of Selumetinib followed by I-131 therapy in iodine refractory patients with differentiated thyroid cancer demonstrating increased iodine uptake following initial treatment with Selumetinib. The primary objective is to assess whether the treatment schedule leads to increased PFS compared to historical control data. PFS has been chosen as the primary endpoint assessing efficacy to allow for proof of concept in this Phase II trial to be obtained in a quick and robust manner and as it is clinically relevant to patients.

### Secondary objectives

- Assess the safety and toxicity of Selumetinib;
- Assess efficacy based on response and overall survival of Selumetinib followed by I-131 therapy;
- Determine the rate of iodine uptake in metastatic lesions in iodine refractory patients with differentiated thyroid cancer treated with Selumetinib.

### Exploratory objectives

- Investigate the role of lesional dosimetry using I-123 SPECT/CT to predict response to I-131 therapy;
- Assess correlation between PBI with absorbed doses delivered and treatment outcome in patients who receive I-131;
- Summarise BRAF, NRAS and TERT gene mutational status, and additional mutational analysis of relevant genes, from tissue and circulating cell-free DNA;
- Assess patient reported quality of life.

### Sample size

Based on previous data, median progression free survival is estimated to be approximately six months, corresponding to 25% of patients alive and progression free at 12 months [5]; a clinically important increase suggesting further investigation of Selumetinib (assuming exponential survival) is deemed to be an increase to 44% (hazard ratio = 0.6). Using a 1-sided 10% significance level, and with 80% power, 38 patients with sufficient radioiodine uptake following Selumetinib therapy are required (allowing for 10% drop out), based on a Case and Morgan two-stage design [27]. Assuming sufficient increase in iodine uptake to warrant I-131 therapy in 60% of patients [21], approximately 60 patients will be recruited in total.

A formal interim analysis will be performed after 21 participants with sufficient iodine uptake following Selumetinib treatment have been recruited and after a minimum of 12 months recruitment. If there is evidence to suggest that 12 month progression free survival is no better than 25% based upon the Nelson-Aalen estimator then the trial may be terminated early for lack of activity.

If none of the first 10 participants treated with Selumetinib has sufficient increase in iodine uptake to progress to I-131 therapy, then the trial may be discontinued. This corresponds to excluding an uptake rate of at least 26% with 95% confidence.

### Eligibility criteria

Inclusion and exclusion criteria can be found in Table 1. Patients with Gilbert’s syndrome (persistent or recurrent hyperbilirubinemia that is predominantly unconjugated in the absence of evidence of haemolysis or hepatic pathology) will be eligible. Eligibility waivers will not be granted in this trial.

**Table 1:** Eligibility criteria

*Inclusion Criteria*	
- Diagnosed with locally advanced or metastatic differentiated thyroid cancer (papillary, follicular, Hürthle cell, or poorly differentiated carcinoma) with at least one measurable lesion as measured by computed tomography (CT) or magnetic resonance imaging (MRI)	
- Participants must have iodine refractory disease defined by one or more of the following: • One or more measurable lesions that do not demonstrate iodine uptake on a previous radioiodine scan (diagnostic uptake or post therapy) OR • One or more measurable lesions that have progressed by RECIST 1.1 criteria within 12 months of I-131 therapy, despite demonstrable radioiodine avidity at the time of that treatment	
- Participants must have radiological progression by RECIST 1.1 criteria within the prior 12 months	
- Measurable disease by RECIST 1.1 criteria. Baseline scan must be completed within 4 weeks prior to the start of treatment.	
- ECOG Performance Status ≤1 and able to tolerate radioiodine therapy	
- Life expectancy of at least 12 weeks	
- Required laboratory values within 14 days of day 1 of treatment: • Adequate thyroid-stimulating hormone (TSH) suppression < 0.5 mU/L • Creatinine clearance > 50 ml/min • Absolute Neutrophil Count ≥1.5 × 109/L (1500 per mm3) • Platelets ≥100 × 109/L (100,000 per mm3) • Haemoglobin > 9.0 g/dL • Serum bilirubin ≤1.5 x upper limit of normal (ULN) • Patients with no liver metastasis must have AST or ALT ≤2.5 x ULN • Patients with liver metastasis must have AST or ALT ≤5 x ULN. If patients have AST or ALT > 3.5 x ULN and ≤ 5 x ULN they must have an ALP ≤ 6 x ULN	
- Able to give informed consent and willing to follow trial protocol.	
- Aged over 18 years or over	
- Female participants of child-bearing potential must have a negative pregnancy test within 24 h prior to starting therapy and agree to use dual methods of contraception for the duration of the trial and 6 months after completing treatment. Male participants must agree to use a barrier method of contraception for the duration of the trial and 4 months after completing treatment, if sexually active with a female of child-bearing potential.	
- Able to swallow Selumetinib/I-131 capsules whole	
*Exclusion criteria*	
- Foci of anaplastic thyroid cancer	
- Able to receive curative surgery or radiation therapy	
- Major surgery (with the exception of surgical placement for vascular access), open biopsy, or significant traumatic injury ≤30 days prior to registration	
- Previous or concurrent cancer distinct in primary site or histology from thyroid cancer within previous 5 years, except for cervical cancer in situ, treated basal cell carcinoma, or squamous cell carcinoma of the skin or superficial bladder tumour	
- Have received or are receiving an IMP or other systemic anticancer treatment within 4 weeks prior to the first dose of study treatment (6 weeks for nitrosoureas, mitomycin, and suramin), or within a period during which the IMP or anticancer treatment has not been cleared from the body (e.g. a period of 5 ‘half-lives’), whichever is the most appropriate and as judged by the investigator	
- Any unresolved toxicity ≥CTCAE Grade 2 from previous anti-cancer therapy, except for alopecia	
- Prior exposure to Tyrosine Kinase, MEK, RAS or RAF inhibitors	
- Known or suspected allergy to Selumetinib or hypersensitivity to Selumetinib or any excipient agents or history of allergic reactions attributed to compounds of similar chemical or biologic composition to Selumetinib	
- Have known or suspected brain metastases or spinal cord compression, unless the condition has been asymptomatic, has been treated with surgery and / or radiation, and has been stable without requiring corticosteroids nor anti-convulsant medications for at least 4 weeks prior to the first dose of study medication.	
- Requiring medication with high iodine content (e.g. amiodarone)	
- Participants who have had a Iodine contrast enhanced CT scan in previous 2 months	
- Ophthalmological conditions as follows: • Intra-ocular pressure > 21 mmHg, or uncontrolled glaucoma (irrespective of intra-ocular pressure) • Current or past history of retinal pigment epithelial detachment (REPD)/central serous retinopathy or retinal vein occlusion	
- Any of the following cardiac conditions • Uncontrolled hypertension (BP ≥150/95 mmHg despite medical therapy) • Acute coronary syndrome within 6 months prior to starting treatment • Uncontrolled angina (Canadian Cardiovascular Society grade II-IV despite medical therapy) • Symptomatic heart failure (NYHA grade II-IV), prior or current cardiomyopathy, or severe valvular heart disease • Prior or current cardiomyopathy including but not limited to the following: • Known hypertrophic cardiomyopathy • Known arrhythmogenic right ventricular cardiomyopathy • Severe valvular heart disease • Left ventricular ejection fraction < 55% measured by echocardiography • Atrial fibrillation with a ventricular rate > 100 bpm on ECG at rest • QTcF > 450 ms or other factors that increase the risk of QT prolongation	
- Participants known to be infected with human immunodeficiency virus (HIV) or hepatitis B (HBV) or C (HCV) virus	
- Any evidence of severe or uncontrolled systemic disease (e.g. unstable or uncompensated respiratory, cardiac, hepatic, or renal disease), active infection, active bleeding (including hepatitis B, hepatitis C, HIV), diatheses or renal transplant.	
- Pregnant or breastfeeding females	
- Male or female patients of reproductive potential and, as judged by the investigator, are not employing an effective method of birth control	
- Have evidence of any other significant clinical disorder or laboratory finding that, as judged by the investigator, makes it undesirable for the patient to participate in the study.	
- Hypersensitivity to bovine or human thyroid stimulating hormone or to any of the following excipients; mannitol, sodium phosphate monobasic, monohydrate, sodium phosphate dibasic, heptahydrate or sodium chloride.	
- Hypersensitivity to I-123 or I-131 or to any of the following excipients; acetic acid, sodium hydroxide, sodium thiosulphate, sodium bicarbonate, sodium chloride, disodium phosphate anhydrous or silica	
- Patients with dysphagia, oesophageal stricture, active gastritis, gastric erosions and peptic ulcer.	
- Have refractory nausea and vomiting, chronic gastrointestinal diseases (e.g., inflammatory bowel disease), suspected reduced gastrointestinal motility or significant bowel resection that would adversely affect the absorption/bioavailability of orally administered study medication.	

### Consent, screening and registration

Written informed consent will be obtained prior to the commencement of all trial assessments, tests and procedures by authorised members of the trial team. Screening investigations will be conducted to confirm eligibility following consent and prior to registration. These investigations will include: CT neck, chest, abdomen and pelvis (without iodinated contrast); echocardiogram/multiple gated acquisition (MUGA); single 12-lead electrocardiogram (ECG); vital signs; ophthalmology examinations (best corrected visual acuity; intraocular pressure and slit lamp fundoscopy); full blood count (FBC); urea and electrolytes (U&Es); liver function tests (LFTs) and thyroid function tests (TFTs); serum pregnancy test for premenopausal women. Eligible patients will then be registered to the trial centrally via the University of Leeds automated 24 h system. Whenever possible, a biopsy of an identified iodine refractory lesion will be obtained following registration; archival tissue from initial thyroidectomy will also be requested.

### Treatment and assessment schedules

Quality of life will be assessed within 28 days of registration for all participants, and tissue samples will be collected within the same timeframe where applicable. Participants will be asked to observe a low iodine diet from two weeks prior to receiving a baseline I-123 SPECT/CT scan which must be completed within 28 days of registration; 0.9 mg recombinant human TSH (rhTSH) will be given via intramuscular administration two days and one day prior to administration of I-123. Prior to day one of treatment participants will have further assessments including repeat blood tests.

Participants will then be treated with 75 mg of oral Selumetinib twice daily for four weeks; doses will be taken approximately 12 h apart. Repeat assessments including haematology and biochemistry will be conducted on day 14 and day 28; where applicable blood samples for cell-free DNA assessment will also be collected. An I-123 SPECT/CT scan at the end of treatment, day 28 (±1 day), will be reviewed centrally. The increase in uptake will be deemed significant if there is presence of any clinically relevant, targeted uptake in a lesion in which there was previously no uptake or there is an increase of 30% or more in uptake in a lesion with evidence of some uptake on the baseline scan. Participants will be asked to recommence the low iodine diet two weeks prior to the post treatment scan and will again be administered 0.9 mg rhTSH two days and one day prior to the scan. Participants will continue to observe the low iodine diet and take Selumetinib whilst the post treatment scan is undergoing review (for a maximum of 18 days). A further two to three SPECT/CT scans will also be acquired and used to predict the radiation dose that may be achieved with radioiodine treatment.

Participants not deemed to have sufficient increase in iodine uptake will cease treatment with Selumetinib, can return to a usual diet and will continue to be followed up for safety purposes until 30 days after completion of Selumetinib treatment. These individuals will receive no further trial treatment.

Participants with sufficient increase of iodine uptake will go on to receive treatment with I-131 and will continue to take Selumetinib until two days after administration of I-131 (if applicable) when the low iodine diet can be discontinued. rhTSH 0.9 mg will be administered two days and one day prior to I-131 therapy. An activity of 5.5 GBq (±10%) I-131 sodium iodide will be administered orally. In the absence of any strong evidence of benefit from a dosimetric approach, it is felt reasonable to stick to empirical activities as per current standard UK practice. However, following I-131 therapy, dosimetry calculations will be made from three to four SPECT/CT scans. A standardised calibration protocol will be used by sites to optimise and standardise SPECT/CT image data across participants. Therefore an essential element of the this trial is the need to set up, for the first time, a network of UK centres able to perform quantitative I-123 and high activity I-131 imaging.

For participants consenting to blood sample collection for PBI analysis, samples will be collected 24 and 144 h post I-131 therapy.

Upon completion of I-131 therapy, participants will be followed up quarterly for the first 12 months and then 6 monthly for the duration of the trial (i.e. until the last patient recruited reaches 12 months follow up) or until evidence of disease progression is confirmed by CT scan. Follow-up assessments will include measurement of thyroglobulin and antithyroglobulin antibodies; thyroid function test; CT neck, thorax, abdomen and pelvis. Quality of life assessments will be repeated at each follow up for the first 12 months, and where applicable blood sample collection for cell-free DNA assessment will occur at three months and six months.

For all participants, TSH suppression to less than 0.1 units should be maintained throughout treatment and follow-up. Also, for all participants (including those not receiving I-131 therapy), assessments at 30 days post end of Selumetinib treatment will include vital signs, 12-lead ECG, haematology, echocardiogram and repeat quality of life measures.

**Fig. 1:**
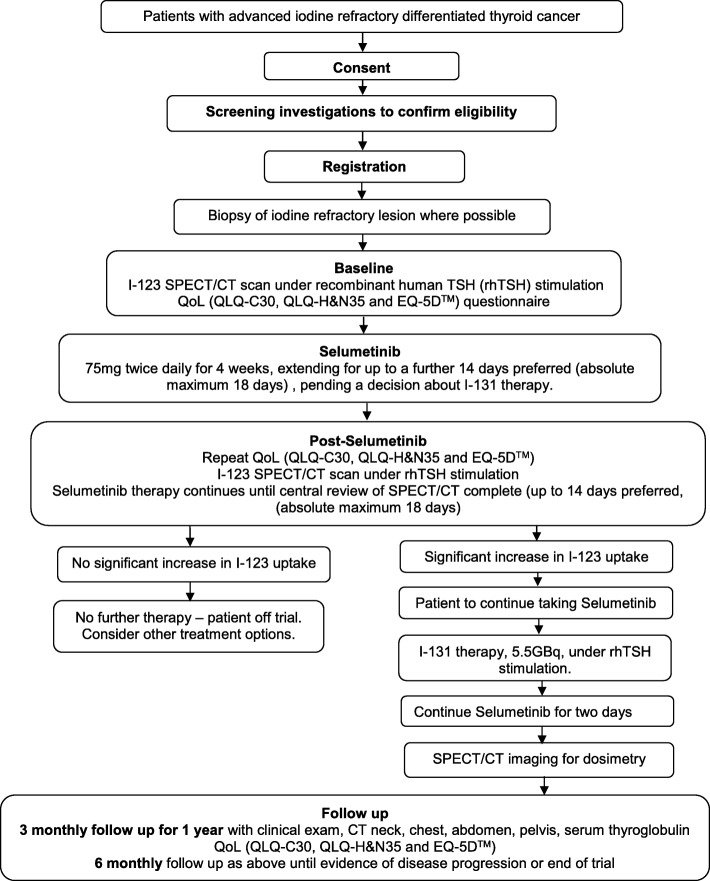
Participant pathway

The participant pathway can be seen schematically in Fig. 1, and Table 2 (SPIRIT diagram) details the assessment schedule.

**Table 2:** Minimum schedule of assessments (SPIRIT diagram)

	Baseline		Treatment				End of Treatment	Follow Up
	Within 14 days prior to registration	Within 28 days of registration	Selumetinib			I-131 Therapy	30 days post end of Selumetinib treatment	3 monthly for first 12 months then 6 monthly for duration of trial^b^
			Day 1	Day 14	Day 28			
Informed consent	X							
Patient details (initials, date of birth, sex)	X							
Medical History	X							
Physical Examination/Vital signs	X		X	X	X		X	X
Ophthalmologic assessment	X							
12-Lead ECG	X		X				X	
Laboratory tests	X		X	X	X		X	X
Pregnancy test	X		X			X	X	X^c^
Echocardiography /MUGA	X		X					
Dispensing of Selumetinib			X					
Tissue collection		X						
CT neck, thorax, abdomen and pelvis	X^a^							X
I-123 SPECT/CT		X			X			
SPECT/CT for Lesion Dosimetry post I-131 therapy						X		
Adverse Events	X		X	X	X	X	X	X^d^
Concomitant Medications	X		X	X	X		X	
Quality of life (QLQ-C30, QLQ-H&N35 and EQ-5D™)		X					X	X^e^
Protein bound iodine blood sample collection						X^f^		
Cell-free DNA blood sample collection			X	X	X		X	X^g^

^a^if CT scans have not been performed within 28 days of starting treatment, an additional CT neck, chest, abdomen, and pelvis must be performed to enable assessment of response and progression-free survival as per RECIST criteria

^b^Participants who do not proceed to radioiodine therapy will be followed up for safety purposes until 30 days after completion of Selumetinib treatment only

^c^Pregnancy test to be done at the first and second 3 monthly follow up visits (3 month and 6 month follow-up)

^d^Adverse reactions only at follow up visits

^e^Quality of life is assessed at baseline, end of treatment and 3 monthly for the first 12 months of follow-up

^f^Collected 24 and 144 h following I-131 dosing

^g^3 and 6 months only

### Outcomes

#### Primary endpoint

The primary endpoint of the trial is PFS at 12 months, reported as the percentage of participants alive and progression-free at 12 months. PFS is calculated from date of registration to first documented evidence of disease progression or death. Participants who have not progressed at the time of analysis will be censored at the last date alive and progression-free.

#### Secondary endpoints

- Safety: Serious adverse events (SAEs) from registration until 30 days post cessation of trial therapy (Selumetinib or I-131 therapy); suspected unexpected serious adverse reactions (SUSARs) and serious adverse reaction (SARs) recorded for all from start of protocol treatment for the lifetime of the trial;
- Toxicity: Adverse reactions (ARs) as graded by common terminology criteria for adverse events (CTCAE) v4.0;
- Overall survival (OS): calculated from date of registration to date of death; censoring will occur at last known date alive for those alive at time of analysis;
- Sufficient iodine uptake: reviewed centrally; sufficient uptake is defined as presence of any clinically relevant uptake in a lesion in which there was previously no uptake or an increase of 30% or more in uptake in a lesion with evidence of some uptake on the baseline scan;
- Radiological response: assessed via CT scans and based on response evaluation criteria in solid tumours (RECIST) v1.1.

#### Exploratory endpoints

- Lesional and whole body dosimetry: As measured from a minimum of three and maximum of four SPECT/CT scans over six days following Selumetinib and I-131;
- BRAF, NRAS and TERT gene mutational status and additional relevant genes: from circulating cell-free DNA, iodine refractory tissue samples and historic tissue samples where available;
- PBI levels from blood samples;
- Quality of life: assessed using QLQ-C30 plus head and neck cancer supplementary questions (H&N35), and EQ-5D™;
- Change in serum thyroglobulin levels: assessed via percentage change from baseline at each time point.

### Analysis

Statistical analysis for final and interim analyses are fully detailed in separate analysis plans. The full analysis set will include all registered participants receiving at least one dose of Selumetinib and will be equivalent to the safety analysis set. The iodine uptake (IU) cohort will include participants from the full analysis set who were deemed to have sufficient I-123 uptake following treatment with Selumetinib. All other participants in the full analysis set will be the non-IU cohort. All efficacy endpoint analyses will be conducted on the IU cohort only, unless otherwise specified in the analysis plan. Safety analyses will be conducted on the safety analysis set with data summarised for each study cohort and overall during initial therapy.

#### Primary endpoint analysis

The proportion of participants alive and progression free at 12 months will be presented with corresponding confidence intervals (CI). The primary analysis will test the hypothesis that PFS in the IU cohort is less than or equal to 25%, against an alternative of 44% based on the Nelson-Aalen estimate of the cumulative hazard function at 12 months, following the methodology of Case and Morgan [27].

#### Secondary endpoint analyses

SAEs, SARs and SUSARs will be reported overall and by treatment period (Selumetinib, I-131) for all participants and for the IU and non-IU cohorts.

The number and proportion of participants experiencing each grade of toxicity will be summarised in the same analysis sets.

OS will be summarised using Kaplan-Meier curves; OS at 12 months and median OS will both be presented alongside CIs.

The proportion of participants with sufficient iodine uptake to progress to I-131 will be presented with corresponding 95% CI. This will also be presented excluding individuals who did not receive Selumetinib for seven days prior to the scan.

The proportion of participants in the IU cohort achieving at least a partial response will be presented with 95% CIs. The proportion of participants in each best response category (complete response, partial response, stable disease, and progressive disease) will also be presented.

#### Exploratory endpoint analyses

The absorbed dose to each lesion, whole body and blood measurements will be presented descriptively alongside measures of uncertainty. Regression modelling will be used to investigate association between these doses and outcome as appropriate. Analogous analyses will explore association between lesional absorbed dose, radiological response and overall survival, as will analyses considering whole body and blood absorbed doses and grades of toxicity.

Gene mutational status will be summarised for all available samples. PFS and response will be summarised by gene mutational status.

PBI measurements will be summarised for all participants receiving I-131 with samples available. The relationship between absorbed dose delivered and treatment outcome will be investigated.

Quality of life will be summarised with 95% CIs adjusting for baseline mean scores. The QLQ-C30 and H&N35 will be summarised for each domain. The proportion of participants scoring each category for the five EQ-5D™ domains will be presented.

Mean change in thyroglobulin from baseline at each time point will be presented with corresponding standard error. Mean change will also be presented by maximum RECIST response. A waterfall plot of maximum change per participant will be presented.

### Trial and data monitoring

A Trial Steering Committee will be convened to periodically review safety data; an independent Data Monitoring and Ethics Committee (DMEC) will review safety data to determine patterns and trends of events which would not be apparent on an individual case basis. Following the interim analysis, the DMEC may request that the trial be terminated for lack of activity if there is evidence to suggest that 12 month PFS is no better than 25% based on the Nelson-Aalen estimator.

## Discussion

The SEL-I-METRY trial will investigate a potential treatment to resensitise radioiodine refractory patients with differentiated thyroid cancer. Current treatment options for this group of patients, the multi-targeted kinase inhibitors Sorafenib and Lenvatinib, need to be given continuously and are associated with significant toxicity and hence may be detrimental to quality of life.

Patients who have radioiodine avid disease have better survival outcomes and thus resensitising refractory patients could lead to improvements in progression-free survival without the need for continuous treatment and the associated toxicity. There are already considerable data regarding the tolerability of the Selumetinib regimen, and a pilot trial found an indication of an increase in iodine uptake. Both toxicity and efficacy will be assessed in this larger Phase II multicentre trial.

If the SEL-I-METRY trial demonstrates that Selumetinib followed by further I-131 NaI is a useful treatment strategy for this group of patients, a randomised trial comparing this approach with other treatments, such as the multi-kinase inhibitors, could be considered to help define the optimal treatment for these patients.

In addition, an essential element of this trial is the need to set up, for the first time, a network of UK centres able to perform quantitative high activity I-131 imaging which is essential for dosimetric calculations. This information will be collected to investigate prediction of response to I-131, presenting an unprecedented opportunity to investigate the role of lesional dosimetry in molecular radiotherapy, leading to greater personalisation of therapy, both with I-131 and with other isotope therapies.

## Trial status

At the time of submission, SEL-I-METRY is open to recruitment.

## Additional file

Additional file 1: Patient Imaging and Dosimetry Protocol. (DOC 19 kb)

Additional file 2: Recommended items to address in a clinical trial protocol and related documents*. (DOC 122 kb)

## Abbreviations

AE: Adverse event
AR: Adverse reaction
CI: Confidence intervals
CTCAE: Common terminology criteria for adverse events
EGC: Electrocardiogram
FBC: Full blood count
GBq: Giga Becquerel
Gy: Gray
I-123: Iodine-123
I-124: Iodine-124
I-131: Iodine-131
IU: Iodine uptake
LFT: Liver function test
MAPKinase: Mitogen-activated protein kinase
MUGA: Multiple gated acquisition
NaI: Sodium iodide
NIS: Sodium iodide symporter
OS: Overall survival
PBI: Protein bound iodine
PET: Positron emission tomography
PFS: Progression free survival
RAI: Radioactive iodine
rhTSH: Recombinant human thyroid stimulating hormone
SAE: Serious adverse event
SPECT/CT: Single photon emission computed tomography/computed tomography
SPIRIT: Standard protocol items: recommendations for interventional trials
SUSAR: Suspected unexpected serious adverse reaction
TFT: Thyroid function tests
TSH: Thyroid stimulating hormone
U&Es: Urea and electrolytes
UK: United Kingdom
